# Communicating treatment risks and benefits to cancer patients: a systematic review of communication methods

**DOI:** 10.1007/s11136-020-02503-8

**Published:** 2020-04-24

**Authors:** L. F. van de Water, J. J. van Kleef, W. P. M. Dijksterhuis, I. Henselmans, H. G. van den Boorn, N. M. Vaarzon Morel, K. F. Schut, J. G. Daams, E. M. A. Smets, H. W. M. van Laarhoven

**Affiliations:** 1 Department of Medical Oncology, Amsterdam University Medical Centers, Cancer Center Amsterdam, University of Amsterdam, P.O. Box 22700, 1100 DE Amsterdam, The Netherlands; 2 Department of Medical Psychology, Amsterdam University Medical Centers, Amsterdam Public Health, University of Amsterdam, Amsterdam, The Netherlands; 3 Amsterdam University Medical Centers, Medical Library, University of Amsterdam, Amsterdam, The Netherlands

**Keywords:** Risk communication, Health-related quality of life, Side effects, Survival, Cancer

## Abstract

**Purpose:**

Cancer patients are increasingly involved in decision-making processes. Hence, clinicians need to inform patients about the risks and benefits of different treatment options in order for patients to make well informed decisions. The aim of this review is to determine the effects of methods of communicating prognostic information about (1) disease progression (survival, progression, recurrence and remission), (2) side effects and complications and (3) health-related quality of life (HRQL) on cognitive, affective and behavioral outcomes in cancer patients.

**Methods:**

A literature search was performed to select articles that were published up to  November 2019 and that examined verbal and/or visual risk communication interventions in an oncological clinical setting.

**Results:**

The search yielded 14,875 studies; 28 studies were ultimately included. For disease progression information, we found that framing affects treatment choice. Furthermore, limiting the amount of progression information in a graphical display could benefit patients’ understanding of risks and benefits. For prognostic information about side effects and complications, precise and defined risk information was better understood than information presented in words. When displaying HRQL data, no consensus was found on which graph type to use.

**Conclusion:**

Great heterogeneity in the results and methodology and in the compared communication formats precluded us from drawing any further conclusions. Practical implications for clinicians are to consider the effects that different types of framing might have on the patient and to not rely exclusively on words to describe risks, but rather include at least some form of numbers or visualization.

**Electronic supplementary material:**

The online version of this article (10.1007/s11136-020-02503-8) contains supplementary material, which is available to authorized users.

## Introduction

In daily clinical practice, many decision making situations occur in which there is no ‘single best treatment option’, since either the medical scientific evidence on the benefit–harm ratio of the options is insufficient, or the ratio is dependent on patients’ values [1]. There is increasing consensus that in these situations, the patient and clinician need to work together and determine what is best for the patient, a process called shared decision-making (SDM) [2, 3].

The clinician however remains responsible for the transfer of probabilistic information to the patient, which is an important step in shared decision-making. If there are multiple treatment options, patients should receive information about disease progression or survival, the risk of side effects and complications, and the impact on health-related quality of life (HRQL) for each option. It is of importance that patients understand the risks and benefits [4], only then can the decision-making process result in the best choice for this particular patient.

However, research shows that correct understanding of relevant outcome information is by no means always achieved in cancer care. For example, studies have shown that breast cancer patients do not fully understand probabilistic information as presented by the clinician [5, 6]. In the case of rectal cancer, only a few patients were found to be able to correctly estimate probabilistic information on treatment outcomes [7]. Clinicians can use different methods to communicate risks, for example, by using words or numbers, by using different types of graphic displays, or by framing the information negatively or positively (in terms of survival or mortality). Based on the review by *Zipkin*, et al*.*, recommendations are available for risk communication in health care, such as avoiding the exclusive use of qualitative risk descriptors (such as ‘many’, or ‘high’), supplementing bar charts or icon arrays to numerical risks, and recognizing that framing and the use of relative risk reductions (RRRs) (see Table 1) influences decision making [8]. However, to date, we do not know to what extent these recommendations apply to the specific context of making decisions about cancer treatment. These are difficult decisions because of the complexity of most treatments. Moreover, the life-threatening nature of the disease induces many emotions [9], which might affect information processing and thus the decision-making process [10]. Furthermore, we do not know how these recommendations might differ for different types of probabilistic information: disease progression, side effects and complication or HRQL [8].

The aim of this systematic review is to evaluate the evidence on the effect of different ways of communicating treatment-related disease progression, side effects and complications and HRQL risk information to cancer patients who have to decide about treatment. Therefore, the effects of different communication methods will be evaluated on three levels: cognitive outcomes (such as patients’ understanding), affective outcomes (such as preference for communication method) and behavioral outcomes (such as treatment choice).

**Table 1:** Examples of verbal and visual methods of communication

Method of communication	Example
*Verbal communication*	
Positive framing	…65 out of 100 patients are alive at 1 year…
Negative framing	…35 out of 100 patients die in 1 year time…
Mixed framing	…65 out of 100 patients are alive at 1 year, 35 out of 100 patients die in 1 year time …
Frequency	…80 out of 100 patients…
Percentage	…80% of patients…
Words	…there is a high risk of…
Absolute risk reduction (ARR)	… the risk of death can be lowered by 3%, from 15 to 12% … (*ARR* = *Event rate 1—Event rate 2*)
Relative risk reduction (RRR)	…the risk of death can be lowered by 20%… (*RRR* = *ARR/Event rate 1*)
Number needed to treat (NNT)	…if 33 patients would be treated, 1 would survive because of the treatment… (*NNT* = *1/ARR)*
Absolute survival benefit (ASB)	…the chance of survival can be increased by 5%, from 76 to 81%…
*Visual communication*	
Line graph	
Bar chart	
Pictograph	
Pie chart	

## Methods

### Search method

A literature search was conducted on the 28th of March 2018 in PubMed, Medline, Embase, PsycINFO and Web of Science. A search update was performed on the 14th of November 2019. Scanning the references of the articles that were initially retained, using citation analysis, helped us to create a set of potentially relevant publications. From this reference set, key concepts for the systematic search were identified. All references from this search had to be retrieved by the final systematic search. Keywords related to the concepts of (surrogate) patients, communication methods (such as graphical, numerical and verbal information), outcomes (such as preference and understanding) and study designs (such as randomized controlled trial and observational studies) were used to search the databases. A broad search strategy was applied (see Online Resource 1). Duplicates were removed and articles were screened on eligibility based on title and abstract using Rayyan [11] by six reviewers independently. Each article was screened in duplicate by two reviewers. The search in March 2018 was screened by three reviewer pairs (JJvK and LvdW, JJvK and IH, NVM and KS) and the search update in November 2019 was screened by LvdW and WD. The same reviewer pairs were used for title and abstract screening and for screening of the full-text articles.

### Inclusion and exclusion criteria

All studies published in English up to November 14, 2019 with the following characteristics were included:

#### Population and context

Studies in which the participants (≥ 18 years) were diagnosed with cancer were included, as were studies that involved healthy surrogate cancer patients (≥ 18 years), i.e., healthy participants answering as if they had cancer. Only studies focusing on risk communication regarding treatment decisions were included; studies on risk communication in cancer screening programs were excluded.

#### Study design

Randomized and nonrandomized controlled trials and cross-sectional and longitudinal observational studies were included. Qualitative/mixed method studies were only included if (1) quantitative data could be extracted in relation to an outcome and (2) a risk communication intervention was offered. Between- and within-subjects studies were included. Case–control and case-series studies were excluded, as were reports, book chapters, conference abstracts and theses.

#### Interventions

Any method of communicating probabilistic information was included. If different communication methods were compared, the same data needed to have been presented in the compared formats. When studies used decision aids as part of an intervention, the (manipulated) characteristics of the risk communication format needed to have been described in a detailed manner.

#### Outcomes

Cognitive outcomes (e.g., patients’ understanding), affective outcomes (e.g., patients’ preference for the format) and behavioral outcomes (e.g., patients’ treatment choice) were included.

### Data extraction

Data regarding study characteristics and outcomes were extracted by LvdW, using an extraction sheet. Outcomes were classified according to *Zipkin *et al*.* using the categories of cognitive, affective and behavioral outcomes [8].

### Quality assessment

For the randomized, between-subjects trials, methodological quality was assessed with a combination of the criteria formulated by the adapted Cochrane Collaboration Consumer and Communication review group [12] and items from the Cochrane Collaboration Tool for Assessing Risk of Bias (Online Resource 2). The scale involves nine items, which were rated as either ‘not fulfilled’ (0), ‘fulfilled’ (1) or ‘not specified’ (0). Studies with positive scores on more than half of the items (> 4.5) were considered as ‘high quality’. Cross-sectional studies using a within-subjects comparison on the outcomes of interest were rated with an adapted version of the Newcastle–Ottawa Scale for Evaluating Cross-sectional/Survey Studies (Online Resource 3). This scale involves nine items, scored with a maximum of two points per item. Studies with scores > 75% of total attainable points were considered ‘high quality’, scores > 50% as ‘moderate quality’ and 50% or less as ‘low quality’ [13]. For qualitative studies, the Critical Appraisal Skills Programme (CASP) Qualitative Checklist [14] was used to assess methodological quality, which consists of ten items scored with a maximum of 20 points in total [15]. The same cut-off scores as for cross-sectional studies were applied [13]. Quality assessment was performed by LvdW who, in case of uncertainty, discussed with a second reviewer (JJvK) until agreement was reached. To ensure a comprehensive review of the literature, assessed methodological quality was not set as an exclusion criterion.

### Data analysis

Studies were subdivided according to the type of type of prognostic information: disease progression, side effects and complications or HRQL. Information on disease progression includes information about survival, progression, remission and recurrence. Verbal and visual communication methods were defined, as shown in Table 1*.* Analysis was performed separately per type of prognostic information. For each information type, studies investigating the same communication methods were compared according to outcomes (cognitive, affective and behavioral).

## Results

**Fig. 1:**
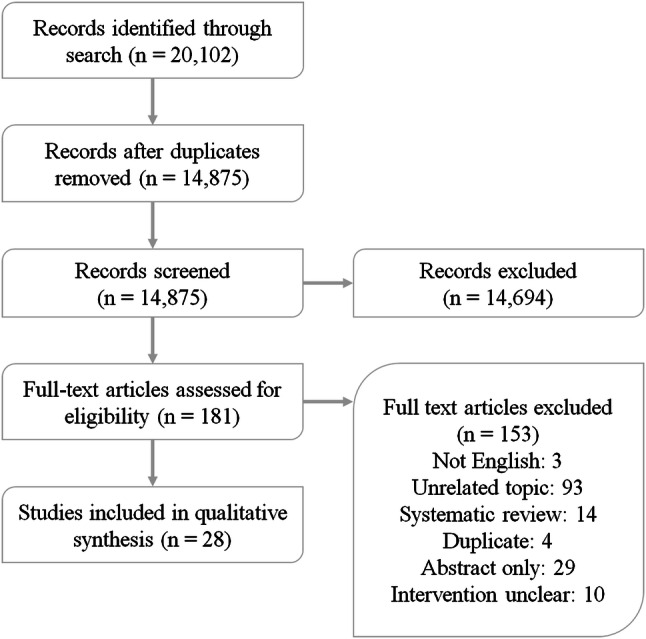
Flow chart of the study inclusion process

The search in PubMed, MEDLINE, PsychINFO, Embase and Web of Science yielded 20,102 articles; removing duplicates resulted in a total of 14,875 articles for screening. Of these, 181 were screened full text, and 28 were included. A summary of the search results can be found in Fig. 1.

Cognitive outcomes were categorized into interpretation accuracy, subjective understanding (including confusion) and cognitive effort. Affective outcomes were categorized into preference for format, satisfaction with format and perceived usefulness (including helpfulness for decision making). All behavioral outcomes (including endorsement of treatment and treatment preference or intentions) were categorized as treatment choice. We found that this outcome was only assessed in studies with disease progression or side effects and complications information.

Seventeen studies investigated probabilistic information regarding disease progression, of which eleven studies included cognitive outcomes (interpretation accuracy, subjective understanding and cognitive effort) [5, 16–25], ten studies included affective outcomes (preference and perceived usefulness) [5, 16, 18, 20, 22, 24–28] and seven studies included behavioral outcomes (treatment choice) [17, 21, 23, 24, 29–31]. One study was not included in the behavioral outcome analysis, because it lacked a control group [26]. Of the seventeen studies on disease progression information, fourteen studies investigated information on survival, two studies on recurrence, one study on progression and one study on remission.

Seven studies investigated probabilistic information regarding side effects or complications, of which four studies included cognitive outcomes (interpretation accuracy) [28, 32–34], six studies included affective outcomes (preference and satisfaction) [28, 32–36] and one study included the behavioral outcome treatment choice [37].

Five studies investigated probabilistic information regarding HRQL, of which four studies included cognitive outcomes (interpretation accuracy and subjective understanding) [38–41] and four studies included affective outcomes (preference and perceived usefulness) [38, 39, 41, 42].

Eleven studies included (cancer) patients [5, 18–22, 26, 28, 36, 38, 42], eight studies included healthy volunteers [16, 17, 23–25, 27, 31, 37] and nine studies included both cancer patients and either healthy volunteers or health-care professionals or all of these [29, 30, 32–35, 39–41].

### Methodological quality

For randomized, between-subjects studies, methodological quality was rated high for seven studies [16, 23, 25, 32, 34, 35, 37] and low for seven studies [17, 21, 24, 27, 30, 33, 40]. Specific scores can be found in Online Resource 4. Items that were often not sufficiently described encompassed whether the method of randomization was truly random, whether allocation was concealed, whether the data analyst was blinded and whether all prespecified outcomes were reported. Only four out of fourteen studies used validated outcome measures to assess one of the outcomes.

For nonrandomized within-subjects cross-sectional studies, methodological quality was rated high for none of the studies, moderate for nine studies [19, 20, 22, 26, 36, 38, 39, 41, 42] and low for four studies [5, 28, 29, 31]. Statistical testing and nonresponse were often reported in insufficient detail. The representativeness and size of the sample were often not satisfactory. All studies used self-report measures, and only two out of eleven studies used validated measures to assess one of the outcomes.

Methodological quality was moderate for the one qualitative study that was included [18].

Because each study assessed and compared another set of communication methods, effects were only described in text if communication methods were compared in more than one study. An overview of the studies’ compared methods and results can be found in Tables 2, 3 and 4.

**Table 2:** Summary of findings for disease progression

Study	Information	Outcome	Design	Compared methods	Findings of interest	Methodological quality
Cognitive outcomes						
Chao 2003^a^ #17	Survival	Interpretation accuracy + Subjective understanding	Randomized cross-sectional, between- -subjects	First: RRR, ARR, ASB, NNT Then: all formats together (RRR + ARR + ASB + NNT)	Participants were significantly less accurate if they had chosen chemotherapy instead of surgery and were presented with RRR in comparison to the other three formats. Confusion ratings were significantly higher when presented with all formats together then with one format only.	Low
Davey 2003 #18	Survival	Interpretation accuracy	Qualitative	*Understanding*: median survival, remaining lifespan, absolute survival, relative survival, conditional survival, graph	Remaining lifespan and absolute survival were correctly understood by most participants (respectively by 18 and 16 out of 26). Median survival was correctly understood by 8 participants and seemed to have often been misinterpreted as the mean. The graph was correctly understood by 6 of 26 and conditional and relative survival data were poorest understood (2 and 1 participant(s), respectively).	Moderate
Davis 2010^a,e^ #19	Survival	Interpretation accuracy	Observational cross-sectional, within-subjects	Bar chart, pictograph, simplified Kaplan–Meier survival curve, textual description (with numbers)	Accuracy rates were significantly lower for the textual description than for the bar chart, pictograph and Kaplan–Meier curve.	Low
Hagerty 2004^b^ #20	Survival	Subjective understanding	Observational cross-sectional, within-subjects	Words, percentage, fraction, pictograph, pie chart, survival (line) graph	Participants found words and numbers easier to understand than visual presentations such as pie charts or graphs.	Moderate
Hamstra 2015^a^ #16	Recurrence	Interpretation accuracy + subjective understanding	Randomized cross-sectional, between-subjects	Pictograph with no numbers labeled in the graph, line graph, pie chart, pictograph and bar chart with only the number of affected individuals labeled, pie chart, pictograph, bar chart with both the number of affected and the number of unaffected individuals labeled	Graphs with both affected and unaffected individuals labeled, resulted in marginally higher accuracy scores than graphs with only the affected individuals labeled (non-significant). There was no significant difference within graphs with 2 numbers (pie chart, pictograph, bar chart) nor within graphs with 1 number (line graph, pie graph, pictograph, bar chart). Significantly less participants answered they would better understand probabilistic information in a pictograph, compared to bar chart, pie graph or line graph.	High
Kang 2018^a,c,c^ #21	Survival	Subjective understanding	Randomized cross-sectional, between-subjects	Words vs. words + pictograph	Participants in the words-only condition, endorsed a significantly higher degree of comprehension than participants in the words and numbers condition.	Low
Kiely 2013^a^ #22	Survival	Subjective understanding	Observational cross-sectional, within-subjects	3 risk scenarios (in words and in a bar plot)—best case, most likely, worst case—vs. median survival (in words)	Significantly more participants found that the three scenarios were easy to understand and improved understanding of survival time compared to median survival.	Moderate
Lobb 1999^b^ #5	Recurrence	Interpretation accuracy	Observational cross-sectional, within-subjects	Absolute risk of relapse, RRR, median 5-year survival, graphical presentation, percentages vs. frequencies, numerical vs. verbal descriptions of risk, positively vs. negatively framed statements	Of participants, 73% interpreted the median correctly. 80% of participants interpreted the graphical presentation correctly. 47% could calculate the RRR correctly based on the ARR and 86% interpreted the absolute risk of relapse correctly. No consistency was found in interpretations of verbally and numerically communicated risks.	Low
Zikmund 2008^a^ #25	Survival	Interpretation accuracy + cognitive effort	Randomized cross-sectional, between-subjects	4-option pictograph, 4-option horizontal bar chart, 2-option pictograph, 2-option horizontal bar chart	Participants who viewed the 2-option pictograph or the 2-option bar chart version were significantly more accurate on a risk reduction question than participants who viewed the 4-option pictograph or the 4-option bar chart. Participants answered more quickly (cognitive effort) when presented with a 2-option graph than when presented with a 4-option graph. 4-option pictographs or 4-option bar charts did not differ significantly in time needed.	High
Zikmund 2010^a^ #24	Survival	Interpretation accuracy + cognitive effort	Randomized cross-sectional, between-subjects	Multi-outcome pictograph (survival and mortality + cause), survival-only pictograph (both pictographs showed 2 treatment options)	Participants who viewed survival-only pictographs were significantly more accurate when reporting the total chance of survival with both combined and hormonal therapy, but not when reporting the incremental chance of survival. Cognitive effort did not differ significantly between the two conditions.	Low
Zikmund 2011^a^ #23	Survival	Interpretation accuracy	Randomized cross-sectional, between-subjects	1 × 4 pictographs at once, 2 × 2 pictographs sequentially	Participants in the sequential condition were significantly more accurate than those in the ‘all at once’ condition.	High
Affective outcomes						
Carey 2018^b^ #28	Survival + remission	Preference	Observational cross-sectional, within-subjects	Words, numbers, both words and numbers	*Remission:* most patients preferred both words and numbers (42%) over only words (30%) or only numbers (10%). 16% had no preference; 05% gave no information; *(5 year) survival*: most patients preferred both words and numbers (43%) over only words (28%), or only numbers (8%). 15% had no preference; 3% did not want any information.	Low
Davey 2003^b^ #18	Survival	Preference	Qualitative	*Framing*: positive, negative, mixed *Presentation*: 100 faces pictograph, numbers and percentages, text only, graph *Stage*: one stage alone, three stages together	25 out of 26 participants preferred positive framing and 1 participant preferred mixed framing. 9 out of 26 participants preferred information on all three stages, 6 preferred to receive information only for their stage.	Moderate
Hagerty 2004^b^ #20	Survival	Preference	Observational cross-sectional, within-subjects	Words, percentage, fraction, pictograph, pie chart, survival (line) graph	Participants preferred words and numbers over visual presentations such as pie charts or graphs.	Moderate
Hamstra 2015^a^ #16	Recurrence	Preference	Randomized cross-sectional, between-subjects	Pictograph with no numbers labeled in the graph, line graph, pie chart, pictograph and bar chart with only the number of affected individuals labeled, pie chart, pictograph, bar chart with both the number of affected and the number of unaffected individuals labeled	Significantly less participants would prefer probabilistic information in a pictograph, rather than in a bar chart, pie graph or line graph.	High
Kiely 2013^a^ #22	Survival	Preference + perceived usefulness	Observational cross-sectional, within-subjects	3 risk scenarios (in a bar plot)—best case, most likely, worst case—only median survival (in words)	48% of participants preferred to receive both three scenarios and median survival, 40% preferred three scenarios only and 5% preferred median only. Participants found the three scenarios significantly more helpful than median survival.	Moderate
Lobb 1999^b^ #5	Recurrence	Preference	Observational cross-sectional, within-subjects	Absolute risk of relapse, RRR, median 5-year survival, graphical presentation, percentages vs. frequencies, numerical vs. verbal descriptions of risk, positively vs. negatively framed statements	43% of participants preferred positively framed risks (eg, ‘chance of cure’), 33% preferred negatively framed messages (eg, ‘chance of relapse’) and 25% had no preference. When comparing frequencies to percentages, 44% of respondents preferred a percentage, 13% preferred a frequency, and the remainder had no preference. When comparing numerical to verbal risks, 53% of participants preferred a numerical description (percentage), 38% preferred a verbal description and the remainder had no preference.	Low
Mazur 1999^b,c^ #26	Survival	Preference	Observational cross-sectional, within-subjects	Words only, numbers only	Of participants having a preference for either numbers or words, 44% had a preference for words only and 56% for numbers only.	Moderate
Studts 2005^a^ #27	Survival	Perceived usefulness	Randomized cross-sectional, between-subjects	RRR, ARR, ASB, NNT	Participants rated the ASB significantly more often as most helpful and most influential than the other three methods (ARR, RRR and NNT).	Low
Zikmund 2008^a^ #25	Survival	Preference	Randomized cross-sectional, between-subjects	4-option pictograph, 4-option horizontal bar chart, 2-option pictograph, 2-option horizontal bar chart	The 4-option and the 2-option pictographs received significantly higher preference scores than the 4-option bar chart.	High
Zikmund 2010^a^ #24	Survival	Preference	Randomized cross-sectional, between-subjects	Multi-outcome pictograph (survival and mortality + cause), survival-only pictograph (both pictographs showed 2 treatment options)	Survival-only pictographs were rated significantly better than multi-outcome pictographs.	Low
Behavioral outcomes						
Chao 2003^a^ #17	Survival	Treatment choice: *Adjuvant chemotherapy vs. No adjuvant chemotherapy (in addition to surgery and tamoxifen)*	Randomized cross-sectional, between-subjects	First: RRR, ARR, ASB, NNT Then: all formats together (RRR + ARR + ASB + NNT)	Participants who received a RRR were significantly more likely to endorse adjuvant chemotherapy. When participants received all four methods of communicating survival benefits of chemotherapy, there were no significant treatment decision differences. Decision confidence did also not differ significantly between conditions.	Low
Kang 2018^a,c,d^ #21	Progression	Treatment choice: *Surgical consultation vs. Surveillance using CT*	Randomized cross-sectional, between-subjects	Words, words + pictograph	When numeric and graphical information (pictograph) was added to descriptive information (words) about a 2-cm renal tumor, participants favored surgical consultation significantly less often compared to when risk information was provided using words only. This effect was not apparent when participants were presented with the scenario of having a 5-cm renal tumor.	Low
McNeil 1982^a^ #29	Survival	Treatment choice: *Surgery vs. Radiation therapy*	Randomized cross-sectional, between-subjects	Positive and negative framing	In the positive framing condition (probability of living), radiation therapy was significantly less often preferred to surgery than in the negative framing condition (probability of dying).	Low
O’Connor 1989^a^ #30	Survival	Treatment choice: *More toxic treatment with higher survival vs. Less toxic treatment with lower survival*	Randomized cross-sectional, between-subjects	Positive framing, negative framing, mixed framing (both positive and negative)	Participants in the negative framing condition considered the more toxic, more effective treatment significantly less desirable (compared to the less effective, less toxic treatment) than those responding in the positive or mixed frames.	Low
Woodhead 2011^a^ #31	Survival	Treatment choice: *Surgery vs. Radiation therapy*	Observational cross-sectional, within-subjects	Positive and negative framing	Participants were categorized on decisional strategy; strategies that were mainly data-driven or driven by personal experience. When participants used scenario data (instead of experience) to inform their decisions and were presented with a positive frame, they were significantly more likely to choose surgery over radiation therapy compared to when presented with a negative frame.	Low
Zikmund 2010^a^ #24	Survival	Treatment choice: *Hormonal therapy vs. Combined therapy (hormonal* + *chemotherapy)*	Randomized cross-sectional, between-subjects	Multi-outcome pictograph (survival and mortality + cause), survival-only pictograph	Participants in the survival-only condition were significantly less likely to say that they preferred combined therapy (hormonal + chemotherapy) to hormonal therapy.	Low
Zikmund 2011^a^ #23	Survival	Treatment choice: *No adjuvant therapy vs. Hormonal therapy vs. Chemotherapy vs. Both chemotherapy and hormonal therapy*	Randomized cross-sectional, between-subjects	1 × 4 pictographs at once, 2 × 2 pictographs sequentially	Higher-numeracy participants were significantly less likely to prefer chemotherapy when in sequential condition vs. in the ‘all at once’ condition. Lower-numeracy participants did not show a difference influenced by communication method.	High

^a^Found at least one significant result between formats, on the discussed outcome

^b^Did not report any statistical analysis on the discussed outcome

^c^Also communicate risk information on complications on certain treatments, besides information on survival/recurrence

^d^Communicates risk information on cancer progression, which could be seen as the opposite of progression-free survival

^e^Did not report statistical testing on this outcome, but test for quality was performed by the reviewers. Post hoc tests revealed a significant difference between the verbal condition and the other three conditions

**Table 3:** Summary of findings for side effects and complications

Study	Outcome	Design	Compared methods	Findings of interest	Methodological quality
Cognitive outcomes					
Carey 2018^b^ #28	Interpretation accuracy	Observational cross-sectional, within-subjects	Percentage, frequency	61% of patients interpreted a percentage on the risk of side effects correctly, 17% interpreted a percentage on the risk of complications correctly, and 65% interpreted a frequency on the risk of side effects correctly.	Low
Knapp 2009 (exp. 1)^a^ #32	Interpretation accuracy	Randomized cross-sectional, between-subjects	Verbal descriptors, percentage, frequency	Participants in the percentage and frequency conditions were more accurate than those in the verbal conditions, this pattern was significantly different from chance for two of three accuracy questions.	High
Knapp 2009II^a^ #34	Interpretation accuracy	Randomized cross-sectional, between-subjects	Verbal descriptors, (absolute) frequency, combination of verbal descriptors and frequency band	Participants in the frequency condition, demonstrated significantly greater accuracy when estimating the likelihood of themselves or the average person having any side effect from taking tamoxifen. They were also significantly more accurate in estimating the likelihood of having (two of four questioned) side effects than the other two formats.	High
Knapp 2013^a^ #33	Interpretation accuracy	Randomized cross-sectional, between-subjects	Frequency, percentage, frequency and percentage combined	There was no significant difference in accuracy between the three conditions, exept for one of six accuracy questions; participants in the percentage condition were more accurate when estimating personal chance of cataracts then in the frequency condition.	Low
Affective outcomes					
Carey 2018^b^ #28	Preference	Observational cross-sectional, within-subjects	Words, numbers, both words and numbers	Most patients preferred both words and numbers (38%) over only words (28%), or only numbers (16%). 16% had no preference and 0.5% did not want any information.	Low
Knapp 2009 (exp. 1) #32	Satisfaction	Randomized cross-sectional, between-subjects	Verbal descriptors, percentage, frequency	There were no significant differences on satisfaction between the three conditions.	High
Knapp 2009II^a^ #34	Satisfaction	Randomized cross-sectional, between-subjects	Verbal descriptors, (absolute) frequency, combination of verbal descriptors and frequency band	Participants in the frequency condition and in the combined condition were significantly more satisfied with the information they received than those in the verbal condition.	High
Knapp 2016 #35	Satisfaction	Randomized cross-sectional, between-subjects (2 × 2)	- Risk expression: numerical only, combined verbal and numerical - Risk qualifier: "will affect…", "may affect…"	There were no significant differences concerning satisfaction between neither the risk expression formats nor the risk qualifiers.	High
Knapp 2013 #33	Preference	Randomized cross-sectional, between-subjects	Frequency, percentage, frequency and percentage combined	53% of the participants preferred the combined format. However there were no significant differences between conditions.	Low
Zomorodbakhsch 2018^b^ #36	Preference	Observational cross-sectional, within-subjects	Words, both words and numbers	Most patients preferred detailed information in words (42%) or detailed information in words with added numbers (32%) over concise, general information in words (5%) or a reference to a booklet (16%).	Moderate
Behavioural outcomes					
Gurich 2018^a^ #37	Treatment choice: *Limb amputation vs. limb salvage*	Randomized cross-sectional, between-subjects	Positive and negative framing	When limb salvage was framed negatively (limb functioning lower than general population), significantly more patients chose amputation than when limb salvage was framed positively (limb functioning higher than with amputation).	High

^a^Found at least one significant result between formats, on the discussed outcome

^b^Did not report any statistical analysis on the discussed outcome

**Table 4:** Summary of findings for quality of life

Study	Outcome	Design	Compared methods	Findings of interest	Methodological quality
Cognitive outcomes					
Brundage 2005^a^ #38	Interpretation accuracy + subjective understanding	Observational cross-sectional, within-subjects	Line graph, line graph with ranges, textual description, side-by-side change (response) bar chart, stacked change (response) bar chart, stacked raw data in bar chart	Both accuracy scores and ease-of-understanding ratings were highest on line graphs showing only the mean scores (no ranges). Textual description showed higher accuracies than the bar chart formats. Both results reached significance.	Moderate
Brundage 2015^b^ #39	Subjective understanding	Randomized cross-sectional, mixed methods,, between-subjects	Simple line graph of mean scores over time, line graph with norms, line graph with confidence intervals, bar chart of average changes, bar chart based on a responder definition (improved, stable, worsened), cumulative distribution function	Within the graphs displaying group-level data, ratings on ease-of-understanding were highest for simple line graphs of mean scores over time.	Moderate
Tolbert, 2018^a^ #40	Interpretation accuracy + subjective understanding	Randomized cross-sectional, between-subjects	Line graphs with lines going up meaning (1) "better" outcomes, (2) "more" of the outcome or (3) lines that were normed to a general population average	Patients interpreted line graphs with lines going up meaning "better" outcomes significantly more accurately than with lines going up meaning "more" of the outcome or "normed" lines. Graphs with lines going up meaning "better" were most often rated as 'very clear' or 'somewhat clear' (non-significant).	Low
Tolbert, 2019^b^ #41	Interpretation accuracy + subjective understanding	Observational cross-sectional, mixed methods, within-subjects	Pie chart, bar chart, icon array	Patients’ interpretation accuracy was highest for pie charts and icon arrays, compared to bar charts. Pie charts were most often rated as ‘very clear’ or ‘somewhat clear’ (no statistical analysis available for patients only).	Moderate
Affective outcomes					
Brundage 2003 #42	Perceived usefulness	Observational cross-sectional, within-subjects	Graphical displays of: nonnumerical trends, mean scores, mean scores with SD's, mean scores described verbally, change in mean scores after six months, individual change scores of two, three or five divisions, normalized/raw scores of 20th and 80th percentile (not clear what kind of graphs the numbers were displayed in)	Participants found graphical displays of mean scores the most useful, but no significant differences were found.	Moderate
Brundage 2005^a^ #38	Perceived usefulness	Observational cross-sectional, within-subjects	Line graph, line graph with ranges, textual description, side-by-side change (response) bar chart, stacked change (response) bar chart, stacked raw data in bar chart	Line graphs displaying mean scores (no ranges) were rated highest on helpfulness and textual descriptions were rated lowest. Helpfulness ratings varied significantly between formats.	Moderate
Brundage 2015^b^ #39	Perceived usefulness	Randomized cross-sectional, mixed methods, between-subjects	Simple line graph of mean scores over time, line graph with norms, line graph with confidence intervals, bar chart of average changes, bar chart based on a responder definition (improved, stable, worsened), cumulative distribution function	When graphs displayed group-level data, ratings on usefulness were highest for simple line graphs of mean scores over time.	Moderate
Tolbert, 2019^b^ #41	Preference	Observational cross-sectional, mixed methods, within-subjects	Pie chart, bar chart, icon array	61% of patient's comments regarding pie charts were coded as positive, 21% of comments regarding bar charts and 35% of comments regarding icon arrays.	Moderate

^a^Found at least one significant result between formats, on the discussed outcome

^b^Did not report any statistical analysis on the discussed outcome

### Probabilistic information on disease progression

Table 2 provides a summary of the results regarding the communication of information about disease progression. Eleven of these studies investigated the effect of different formats on cognitive outcomes. Based on three studies, of which two were of high methodological quality and one of low, graphs were better understood (interpretation accuracy and cognitive effort) when there was less information for the participant to process at one time (in one graph or in different graphs presented at once), compared to more information [23–25]. One study of low quality supported this conclusion for verbal formats, by showing higher confusion rates when different formats were presented all at once, compared to separate presentation [17]. Contradictory results were found when verbal information was compared to graphical information; two studies found higher subjective understanding for words compared to graphs (moderate quality) [20] or compared to graphs accompanied by words (low quality) [21]. However, one study of moderate quality found that graphs were objectively better understood than words [19]. No further conclusions could be drawn on cognitive outcomes because of heterogeneity in the compared communication formats.

Ten studies included affective outcomes, of which nine investigated a preference for formats and two investigated the perceived usefulness of the formats. Although there was much variation in compared formats, two studies, both of high methodological quality, did compare pictographs (among other formats) with bar charts [16, 25]. However, these two studies found conflicting results: one found bar charts to be preferred over pictographs [16] and the other found pictographs to be preferred over bar charts [25]. Two studies of low and moderate quality found positive framing to be preferred over negative framing, although none performed statistical analysis on this effect [5, 18]. When comparing words to numbers, three studies, of low and moderate quality, found conflicting results, of which none performed statistical analysis on this comparison [5, 26, 28].

Seven studies investigated the behavioral outcome treatment choice. Different treatment options were presented in these studies, ranging from very specific (radiotherapy) to more general descriptions of treatment (an unknown treatment that is more toxic but also has higher survival rates). In all but one study there were only two possible options [23]. Three studies investigated the differences in treatment choice when information was framed positively (in terms of survival), negatively (in terms of death) or both (mixed frame) [29–31]. Two of these, both of low methodological quality, found that surgery (higher chance of survival, but risk of perioperative death) was more frequently chosen (instead of radiotherapy—lower chance of survival and more side effects) when information was framed positively instead of negatively [29, 31]. When patients in another low-quality study had to choose between a treatment that was more effective, but more toxic, and one that was less effective and less toxic, the first was less preferred in a negative frame (compared to positive and mixed) [30].

### Probabilistic information on side effects and complications

Table 3 provides a summary of the results regarding information about side effects and complications. Seven studies investigated the effects of communication methods on side effects and complications information [28, 32–37], of which four investigated the cognitive outcome interpretation accuracy, six the affective outcomes preference or satisfaction and one the behavioral outcome treatment choice. Two studies of high methodological quality compared accuracies for risk information in words to percentages and/or frequencies. These studies found that more precise risk information about side effects (percentage/frequency) was superior to information in words [32, 34]. When comparing frequencies to percentages, no clear effect was found. One study of low quality only found a difference on one of six check questions, showing higher accuracies for percentages [33].

Concerning satisfaction with and preferences for communication methods, most studies did not find a significant difference between formats. However, one study of high quality found that communicating frequencies instead of (solely) verbal risk information can contribute significantly to patient satisfaction [34]. Two other studies of low and moderate quality found a description in both words and numbers and a (detailed) description in words only to be most preferred [28, 36]. These two studies did however not report statistical analysis on these preferences.

### Probabilistic information on health-related quality of life

Table 4 provides a summary of the results regarding HRQL information. Four studies compared different methods of communicating HRQL information on cognitive outcomes three looked at interpretation accuracy and four looked at subjective understanding. One study of moderate methodological quality found that basic line graphs were best understood (objectively and subjectively; compared to textual descriptions, line graphs with ranges and several different bar chart formats) [38]. Another moderate-quality study found simple line graphs to score highest on ease of understanding, but did not report any statistical testing on this outcome [39]. With respect to the directionality of line graphs, a study of low quality found lines going up meaning better outcomes, to be interpreted more accurately than lines going up meaning more of the outcome or normed lines [40]. A third study of moderate quality, however, found pie charts to be best understood (objectively and subjectively, compared to bar charts and icon arrays), but did not report statistical testing on this outcome for patients separately [41].

Affective outcomes for HRQL data were compared in four studies. Three studies measured perceived usefulness and one study measured preference for communication method. Three studies found that simple line graphs displaying mean scores were perceived as most useful [38, 39, 42], of which only one study, of moderate quality, reported a significant difference between formats: line graphs displaying mean scores were perceived as most helpful (compared to line graphs with ranges, textual descriptions and various bar chart formats) [38]. Another study, however, reported pie charts to be most positively commented on, compared to bar charts and icon arrays [41].

## Discussion

In this review, we summarized the literature on methods of communicating probabilistic information in oncological treatment decision-making processes.

For communication of disease progression information, we found that the type of framing has an influence on treatment choice. This has also been observed in communication in general health care [8]; positive framing (in terms of survival instead of mortality) may increase acceptance of treatments. However, in this review, we could not draw any conclusions regarding the direction of the effect due to the incomparability of treatment choices and to the low methodological quality of the studies.

Furthermore, we found that limiting the amount of survival information that the patient has to process at once in a graphical display could benefit the patients’ understanding. There is growing evidence supporting the so-called ‘less is more’ approach in the field of decision-making; simpler forms of communication can make it easier for patients to use the information during decision making [24]. Additionally, it has been argued that the complexity of information may contribute to patients’ experience of uncertainty [43].

We found that precise and defined risk information (e.g., percentages) about side effects was better understood than verbal information. Here, however, a potential source of bias could exist in the way that outcomes were measured. To prevent recall bias, the risk format used in the outcome assessment should not be similar to the format used as an intervention. For example, comparing percentages to another format would not be completely ‘fair’ when the answer to an accuracy question has to be stated in percentages, as is the case in at least two of three accuracy studies. However, finding a suitable assessment format that will not be influenced by recall might be challenging in these cases.

For display of HRQL information, we did not find consensus among the included studies on which type of graphs to use. Whereas line graphs and pie charts seemed to result in better cognitive and affective outcomes, these results were only based on one significant result each. While a previous review in general health care [8] recommends using icon arrays or bar charts to display outcome information, recent studies on the display of HRQL information specifically, suggested using pie charts [44, 45]. Another issue is the direction of display of HRQL data when using bar or line graphs. Where other literature in HRQL research—beyond the scope of this review—recommends the graphical display direction of better = higher [45, 46], we did not find enough evidence—one study—to recommend on the direction of HRQL graphs [40].

Great heterogeneity was found not only in the study results but also in methodology and in the compared communication formats. This precludes us from stating separate practice recommendations for the three different types of risk information (disease progression, side effects and complications and HRQL). Most importantly, several studies investigated the same communication methods and found different effects on outcomes. Therefore, a meta-analysis would be of great help. However, for meta-analysis to be possible, the heterogeneity in methodology and compared risk formats needs to be less substantial.

Furthermore, the overall methodological quality of most studies was found to be moderate, which was largely due to the lack of details to properly assess the risk of bias. Additionally, validated measures were not frequently used to assess the analyzed outcomes. The latter may be because few validated measures exist that are applicable to multiple risk information situations. Notably, the adapted quality grading criteria may not have been perfectly suited to evaluate the studies as designed and was only performed by a single author, except where that author requested input from a second author.

In addition to the moderate overall quality and the heterogeneity of studies, the following aspects may also have influenced the outcomes of this review. First, we included studies in which participants were not always cancer patients themselves, but for example students, patients in an outpatient clinic or website visitors. The hypothetical scenario presented to participants could have been less relevant to this group in comparison to patients. Furthermore, by including students, there is a potential effect of numeracy and graph literacy levels, possibly resulting in higher understanding among these participants. We therefore suggest that numeracy and graph literacy be assessed in future research on the subject. We also suggest that social and cultural background variables be assessed in future research, to be able to describe their influence on risk communication.

Despite the above, when combining the results on all three information types, we can provide two main suggestions for cancer clinical practice. First, we recommend that clinicians treating cancer patients consider the effects that positive and negative framing might have on the treatment choice of the patient. Second, we suggest that clinicians not only use words when describing risks but at least also use some form of numbers or visualization to discuss risk. Future researchers should take numeracy and graph literacy effects into account, carefully choose their measures and describe the procedures used in more detail.

## Electronic supplementary material

Below is the link to the electronic supplementary material.

Supplementary file1 (DOCX 18 kb)

Supplementary file2 (DOCX 16 kb)

Supplementary file3 (DOCX 15 kb)

Supplementary file4 (DOCX 24 kb)
