# Supplementary material for: Communicating treatment risks and benefits to cancer patients: a systematic review of communication methods
Source: Qual Life Res. 2020 Apr 24;29(7):1747–66. doi: 10.1007/s11136-020-02503-8 (PMC7295838; doi:10.1007/s11136-020-02503-8)
Supplement: Supplementary file 1 — Supplementary file1 (DOCX 18 kb) [file 11136_2020_2503_MOESM1_ESM.docx]

ESM 1: **Search strategy**

|  | **Ovid MEDLINE(R) Epub Ahead of Print, In-Process & Other Non-Indexed Citations, Ovid MEDLINE(R) Daily and Ovid MEDLINE(R) <1946 to Present> Search date: 28 March 2018** |
| --- | --- |
| **#** | **Searches** |
| 1 | (tumor or neoplasm? or cancer? or malignan* or oncol* or carcinoma or sarcoma).ab,kf,ti. |
| 2 | (communic* or presenting or presentation or presented or consultation or consult or consulted).ab,kf,ti. |
| 3 | ((participant? or client? or subject? or patient? or volunteer?) adj3 decision?).ab,kf,ti. |
| 4 | and/1-3 [patients' decision] |
| 5 | patient satisfaction/ |
| 6 | (patient? adj10 (satisfied or satisfaction or prefer*)).ab,kf,ti. |
| 7 | or/5-6 |
| 8 | consumer health information/ |
| 9 | (graph? or pictograph? or chart? or array? or information).ab,kf,ti. |
| 10 | or/8-9 |
| 11 | and/1-2,7,10 [patients' satisfaction with provided information] |
| 12 | "patient education as topic"/ |
| 13 | ((participant? or client? or subject? or patient? or volunteer?) adj10 (comprehen* or underst*)).ab,kf,ti. |
| 14 | or/12-13 |
| 15 | and/1-2,10,14 [patients'understanding of provided information] |
| 16 | (eq5 d or eq5d or eq 5d or hrqol or qol or (life adj2 quality) or shortform or short form or bref or whoqol or sf36 or sf 36 or sf12 or sf 12 or sf8 or sf 8 or (fact adj2 (score? or scale? or inventor* or question*)) or qlq or eortc or vas or visual analogue scale or manchester short assessment).ab,kf,ti. |
| 17 | and/1-2,10,16 [patients' perceived qol] |
| 18 | or/4,11,15,17 |
|  |  |
|  |  |
|  | **Ovid Embase Classic+Embase <1947 to 2018 March 27> Search date: 28 March 2018** |
| **#** | **Searches** |
| 1 | (tumo?r or neoplasm? or cancer? or malignan* or oncol* or carcinoma or sarcoma).ab,kw,ti. |
| 2 | (communic* or presenting or presentation or presented or consultation or consult or consulted).ab,kw,ti. |
| 3 | ((participant? or client? or subject? or patient? or volunteer?) adj3 decision?).ab,kw,ti. |
| 4 | and/1-3 [patients' decision] |
| 5 | patient satisfaction/ |
| 6 | (patient? adj10 (satisfied or satisfaction or prefer*)).ab,kw,ti. |
| 7 | or/5-6 |
| 8 | consumer health information/ |
| 9 | (graph? or pictograph? or chart? or array? or information).ab,kw,ti. |
| 10 | or/8-9 |
| 11 | and/1-2,7,10 [patients' satisfaction with provided information] |
| 12 | patient education/ |
| 13 | ((participant? or client? or subject? or patient? or volunteer?) adj10 (comprehen* or underst*)).ab,kw,ti. |
| 14 | or/12-13 |
| 15 | and/1-2,10,14 [patients'understanding of provided information] |
| 16 | (eq5 d or eq5d or eq 5d or hrqol or qol or (life adj2 quality) or shortform or short form or bref or whoqol or sf36 or sf 36 or sf12 or sf 12 or sf8 or sf 8 or (fact adj2 (score? or scale? or inventor* or question*)) or qlq or eortc or vas or visual analogue scale or manchester short assessment).ab,kw,ti. |
| 17 | and/1-2,10,16 [patients' perceived qol] |
| 18 | or/4,11,15,17 |
|  |  |
|  |  |
|  | **Ovid PsycINFO <1806 to March Week 3 2018> Search date: 28 March 2018** |
| **#** | **Searches** |
| 1 | (tumo?r or neoplasm? or cancer? or malignan* or oncol* or carcinoma or sarcoma).ab,id,ti. |
| 2 | (communic* or presenting or presentation or presented or consultation or consult or consulted).ab,id,ti. |
| 3 | ((participant? or client? or subject? or patient? or volunteer?) adj3 decision?).ab,id,ti. |
| 4 | and/1-3 [patients' decision] |
| 5 | client satisfaction/ |
| 6 | (patient? adj10 (satisfied or satisfaction or prefer*)).ab,id,ti. |
| 7 | or/5-6 |
| 8 | (graph? or pictograph? or chart? or array? or information).ab,id,ti. |
| 9 | and/1-2,7-8 [patients' satisfaction with provided information] |
| 10 | patient education/ |
| 11 | ((participant? or client? or subject? or patient? or volunteer?) adj10 (comprehen* or underst*)).ab,id,ti. |
| 12 | or/10-11 |
| 13 | and/1-2,8,12 [patients'understanding of provided information] |
| 14 | (eq5 d or eq5d or eq 5d or hrqol or qol or (life adj2 quality) or shortform or short form or bref or whoqol or sf36 or sf 36 or sf12 or sf 12 or sf8 or sf 8 or (fact adj2 (score? or scale? or inventor* or question*)) or qlq or eortc or vas or visual analogue scale or manchester short assessment).ab,id,ti. |
| 15 | and/1-2,8,14 [patients' perceived qol] |
| 16 | or/4,9,13,15 |

Article title: Communicating treatment risks and benefits to cancer patients: a systematic review of communication methods.

Journal: Quality of Life Research

Author names: L.F. van de Water^1,2^, J. J. van Kleef^1,2^, I. Henselmans^2^, H.G. van den Boorn^1^, N.M. Vaarzon Morel^1^, K. F. Schut^1^, J. G. Daams^3^, E.M.A Smets^2^, H.W.M. van Laarhoven^1^*

1. *Amsterdam University Medical Centers, Cancer Center Amsterdam, Department of Medical Oncology, University of Amsterdam, Amsterdam, the Netherlands*
2. *Amsterdam Public Health, Amsterdam University Medical Centers, Department of Medical Psychology, University of Amsterdam, Amsterdam, the Netherlands*
3. *Amsterdam University Medical Centers, Medical Library, University of Amsterdam, Amsterdam, the Netherlands.*

Corresponding author: H.W.M. van Laarhoven, h.vanlaarhoven@amsterdamumc.nl
