# Supplementary material for: Communicating treatment risks and benefits to cancer patients: a systematic review of communication methods
Source: Qual Life Res. 2020 Apr 24;29(7):1747–66. doi: 10.1007/s11136-020-02503-8 (PMC7295838; doi:10.1007/s11136-020-02503-8)
Supplement: Supplementary file 2 — Supplementary file2 (DOCX 16 kb) [file 11136_2020_2503_MOESM2_ESM.docx]

| ESM2: **Adapted version of the Cochrane Collaboration Tool for Assessing Risk of Bias** |
| --- |
| 1. Was the method of randomization adequate, i.e. truly random?*^a^ 2. Was allocation concealed, i.e., could allocation to conditions have been influenced?*^a^ |
| 1. Was the data analyst blinded (i.e., was the code only broken after conclusions were drawn)?^a^ 2. Were groups compared at baseline on at least one potential determinant of the outcomes (e.g., socio-demographics, medical characteristics and primary outcomes) and were the analyses properly controlled for differences?* 3. Was analysis done according to the intention-to-treat principle (i.e., were all allocated subjects included in the analysis, for example by data imputation)?* 4. Were validated measures used to assess at least one outcome of relevance to this review (i.e., by well-known questionnaires or reliable coding schemes)?* 5. Was the study sufficiently powered?* The fairly liberal criterion of Coyne et al. [95] was adopted, which means that based on a power of 55% and a medium effect size (d = 0.05), a sample size of more than 35 per condition is required, i.e., at least 35 respondents should be participating at the first follow-up assessment or, if applicable, for at least 35 respondents recordings of the consultation should be available. 6. Have all pre-specified (primary and secondary) outcomes been reported? 7. Does the study appears to be free of other bias? (i.e., bias due to problems not covered elsewhere in the checklist) |
| * Among the criteria of Cochrane Collaboration Consumer and Communication review group, adapted by Henselmans et al. (1)  ^a^ Considered fulfilled when called random, concealed or blinded by authors or when the text explicitly refers to such methods (such as mention of ‘sealed opaque envelopes’, a cover story for patients in the control group or separate consent forms).  ^b^ With respect to the multiple criteria of blinding, we decided to exclude the criterion of blinding the doctor, the patient and the outcome assessor. In most studies, blinding of the ‘doctor’ or the ‘patient’ was not possible due to the nature of the intervention. Also in most studies, patients themselves were among the outcome assessors. |

REFERENCES

1. Henselmans I, de Haes HC, Smets EMJPO. Enhancing patient participation in oncology consultations: a best evidence synthesis of patient‐targeted interventions. 2013;22(5):961-77.

Article title: Communicating treatment risks and benefits to cancer patients: a systematic review of communication methods.

Journal: Quality of Life Research

Author names: L.F. van de Water^1,2^, J. J. van Kleef^1,2^, I. Henselmans^2^, H.G. van den Boorn^1^, N.M. Vaarzon Morel^1^, K. F. Schut^1^, J. G. Daams^3^, E.M.A Smets^2^, H.W.M. van Laarhoven^1^*

1. *Amsterdam University Medical Centers, Cancer Center Amsterdam, Department of Medical Oncology, University of Amsterdam, Amsterdam, the Netherlands*
2. *Amsterdam Public Health, Amsterdam University Medical Centers, Department of Medical Psychology, University of Amsterdam, Amsterdam, the Netherlands*
3. *Amsterdam University Medical Centers, Medical Library, University of Amsterdam, Amsterdam, the Netherlands.*

Corresponding author: H.W.M. van Laarhoven, h.vanlaarhoven@amsterdamumc.nl
