# Supplementary material for: Communicating treatment risks and benefits to cancer patients: a systematic review of communication methods
Source: Qual Life Res. 2020 Apr 24;29(7):1747–66. doi: 10.1007/s11136-020-02503-8 (PMC7295838; doi:10.1007/s11136-020-02503-8)
Supplement: Supplementary file 3 — Supplementary file3 (DOCX 15 kb) [file 11136_2020_2503_MOESM3_ESM.docx]

## ESM 3: Adapted Newcastle-Ottawa Scale for Evaluating Cross-Sectional/Survey Studies

**Maximum score: 16 points**

1. Clearly Stated Aim (max 2)
2. The question addressed is precise and relevant in light of the available literature **
3. The question addressed is described, but not with sufficient precision *
4. Subject Selection (max 2)
   1. *Representativeness of the Sample*
      1. Truly representative of the average in the target population (all subjects or random sampling) (including if only one institution) **
      2. Somewhat representative of the average in the target population (non-random sampling) *
      3. Selected group of users
      4. No description of the sampling strategy
   2. *Sample Size* (max 2)
      1. Justified and satisfactory **
      2. Justified, not satisfactory or satisfactory, not justified *
      3. Not justified
   3. *Non-respondents* (max 2)
      1. Response rate is assessed^1^ and satisfactory^2^ (>70%)*.* Comparability between respondents and non-respondents characteristics is assessed^3^ **
      2. Two of three *
      3. One or none of three *
5. Comparability (max 2)
   1. *Confounding Factors are Controlled. (max 1)*
6. The study controls for potential confounder(s). *
   1. *When The Subjects in Different Outcome Groups are Comparable, Based on Study Design or Analysis. (max 1)*
7. The study subjects in different groups are comparable, based on design or analysis. *
8. The study subjects in different groups are NOT comparable, based on design or analysis.
9. Not applicable. No group comparisons are made. = -1 total point
10. Outcome *(max 6)*
11. *Assessment of the Outcome (max 2)*
12. Independent blind assessment **
13. Record linkage **
14. Self-report *
15. No description
16. *Measurement of the outcome (max 2)*
    - 1. Validated measurement tool **
      2. Non-validated measurement tool, but the tool or method is available or described *
      3. No description of the measurement tool or method.
17. *Statistical Test (max 2)*
18. The statistical test used to analyze the data is clearly described and appropriate, and the measurement of association is presented, including confidence intervals and probability level (p value). (All present) **
19. The statistical test used to analyze the data is clearly described and appropriate, and the measurement of association is presented, including confidence intervals and probability level (p value). (Two of three present) *
20. The statistical test is not appropriate, not described, or incomplete.
21. Not applicable: qualitative study design. = -1 total point

* Assign 1 point

** Assign two points

Article title: Communicating treatment risks and benefits to cancer patients: a systematic review of communication methods.

Journal: Quality of Life Research

Author names: L.F. van de Water^1,2^, J. J. van Kleef^1,2^, I. Henselmans^2^, H.G. van den Boorn^1^, N.M. Vaarzon Morel^1^, K. F. Schut^1^, J. G. Daams^3^, E.M.A Smets^2^, H.W.M. van Laarhoven^1^*

1. *Amsterdam University Medical Centers, Cancer Center Amsterdam, Department of Medical Oncology, University of Amsterdam, Amsterdam, the Netherlands*
2. *Amsterdam Public Health, Amsterdam University Medical Centers, Department of Medical Psychology, University of Amsterdam, Amsterdam, the Netherlands*
3. *Amsterdam University Medical Centers, Medical Library, University of Amsterdam, Amsterdam, the Netherlands.*

Corresponding author: H.W.M. van Laarhoven, h.vanlaarhoven@amsterdamumc.nl
