# Supplementary material for: Communicating treatment risks and benefits to cancer patients: a systematic review of communication methods
Source: Qual Life Res. 2020 Apr 24;29(7):1747–66. doi: 10.1007/s11136-020-02503-8 (PMC7295838; doi:10.1007/s11136-020-02503-8)
Supplement: Supplementary file 4 — Supplementary file4 (DOCX 24 kb) [file 11136_2020_2503_MOESM4_ESM.docx]

ESM 4: **Methodological quality assessment**

**Methodological quality for randomized cross-sectional, between-subjects studies**

| **Study** | **Random** | **Allocation concealed** | **Blind data analyst** | **Groups compared** | **Intention to treat principle** | **Validated measures** | | **Sufficiently powered** | **Selective reporting** | **Other bias** | **Total** | **Quality** |
| --- | --- | --- | --- | --- | --- | --- | --- | --- | --- | --- | --- | --- |
| Chao 2003 #17 | - | - | - | 1 | 1 | | 0 | 1 | - | 1 | 4 | Low |
| Gurich 2018 #37 | 1 | 1 | - | 1 | 1 | | 0 | 1 | - | 1 | 6 | High |
| Hamstra 2015 #16 | - | - | - | 1 | 1 | | 1 | 1 | - | 1 | 5 | High |
| Kang 2018 #21 | 1 | 0 | - | 1 | 0 | | 0 | 1 | - | 1 | 4 | Low |
| Knapp 2016 #35 | 1 | 1 | - | 1 | 1 | | 0 | 1 | - | 1 | 6 | High |
| Knapp 2009II #34 | 1 | 1 | - | 1 | 0 | | 0 | 1 | - | 1 | 5 | High |
| Knapp 2013 #33 | 1 | 1 | - | 1 | 0 | | 0 | 1 | - | 0 | 4 | Low |
| Knapp 2009 #32 | 1 | 1 | - | 1 | 1 | | 0 | 1 | - | 0 | 5 | High |
| O’Connor 1989 #30 | - | - | - | 1 | 0 | | 0 | 1 | - | 1 | 3 | Low |
| Studts 2005 #27 | - | - | - | 1 | 1 | | 0 | 1 | - | 1 | 4 | Low |
| Tolbert 2018 #40 | - | - | - | 1 | 1 | | 0 | 1 | - | 1 | 4 | Low |
| Zikmund 2008 #25 | 1 | - | - | 1 | 0 | | 1 | 1 | - | 1 | 5 | High |
| Zikmund 2010 #24 | - | - | - | 1 | 0 | | 1 | 1 | - | 1 | 4 | Low |
| Zikmund 2011 #23 | 1 | - | - | 1 | 0 | | 1 | 1 | - | 1 | 5 | High |

*Items were rated as either ‘not fulfilled’ (0), ‘fulfilled’ [1] or ‘not specified’ (-). Quality is considered high if more than half (>6) of the applicable criteria were fulfilled.*

| **Study** | **Clearly stated aim** | **Representativeness of sample** | **Sample size** | **Non-respondents** | **Confounding factors** | **Comparable groups** | **Assessment of outcome** | **Measurement of outcome** | **Statistical test** | **Total score** | **Quality** |
| --- | --- | --- | --- | --- | --- | --- | --- | --- | --- | --- | --- |
| Carey 2018 #28 | 2 | 1 | 1 | 1 | 0 | -1 total | 1 | 1 | 0 | 7/15=47% | Low |
| Brundage 2003 #42 | 2 | 2 | 0 | 1 | 1 | 1 | 1 | 1 | 1 | 10/16=63% | Moderate |
| Brundage 2005 #38 Brundage 2015 | 2 | 2 | 2 | 1 | 1 | -1 total | 1 | 1 | 1 | 11/15=73% | Moderate |
| Brundage 2015 #39 | 2 | 1 | 2 | 1 | 1 | -1 total | 1 | 1 | 0 | 9/15=60% | Moderate |
| Davis 2010 #19 | 1 | 0 | 2 | 1 | 0 | -1 total | 1 | 1 | 2 | 8/15=53% | Moderate |
| Hagerty 2004 #20 | 1 | 1 | 1 | 2 | 0 | -1 total | 1 | 2 | 1 | 9/15=60% | Moderate |
| Kiely  2013 #22 | 2 | 1 | 2 | 1 | 0 | -1 total | 1 | 0 | 2 | 9/15=60% | Moderate |
| Lobb  1999 #5 | 1 | 1 | 1 | 1 | 0 | -1 total | 1 | 1 | 0 | 6/15=40% | Low |
| Mazur 1999 #26 | 2 | 1 | 1 | 1 | 0 | -1 total | 1 | 1 | 1 | 8/15=53% | Moderate |
| McNeil  1982 #29 | 1 | 1 | 1 | 1 | 0 | 0 | 1 | 1 | 0 | 6/16=38% | Low |
| Tolbert 2019 #41 | 2 | 1 | 1 | 1 | 1 | -1 total | 1 | 1 | 1 | 9/15=60% | Moderate |
| Woodhead2011 #31 | 2 | 0 | 1 | 1 | 1 | 0 | 1 | 1 | 1 | 8/16=50% | Low |
| Zomorodbakhsch 2018 #36 | 2 | 1 | 1 | 1 | 1 | -1 total | 1 | 2 | 0 | 9/15=60% | Moderate |

**Methodological quality for non-randomized cross-sectional, within-subjects studies**

*Items were rated as either ‘not fulfilled’ (0), ‘partially fulfilled’(1), ‘fulfilled’(2) or ‘non applicable’(-1 total). Studies with scores >75% of total attainable points were considered as ‘high quality’, scores >50% as ‘moderate quality’ and 50% or less as ‘low quality’.*

**Methodological quality for qualitative studies**

| **Study** | **Clear statement of aims** | **Qualitative methodology appropriate** | **Appropriate design** | **Appropriate recruitment strategy** | **Data collection** | **Relationship considered** | **Ethical issues considered** | **Data analysis** | **Statement of findings** | **Valuable** | **Total** | **Quality** |
| --- | --- | --- | --- | --- | --- | --- | --- | --- | --- | --- | --- | --- |
| Davey 2003 | 1 | 1 | 1 | 2 | 1 | 0 | 1 | 1 | 2 | 1 | 11/20 = 55% | Moderate |

*Items were rated as either ‘not fulfilled’ (0), ‘partially fulfilled’(1) or ‘fulfilled’(2). Studies with scores >75% of total attainable points were considered as ‘high quality’, scores >50% as ‘moderate quality’ and 50% or less as ‘low quality’.*

Article title: Communicating treatment risks and benefits to cancer patients: a systematic review of communication methods.

Journal: Quality of Life Research

Author names: L.F. van de Water^1,2^, J. J. van Kleef^1,2^, I. Henselmans^2^, H.G. van den Boorn^1^, N.M. Vaarzon Morel^1^, K. F. Schut^1^, J. G. Daams^3^, E.M.A Smets^2^, H.W.M. van Laarhoven^1^*

1. *Amsterdam University Medical Centers, Cancer Center Amsterdam, Department of Medical Oncology, University of Amsterdam, Amsterdam, the Netherlands*
2. *Amsterdam Public Health, Amsterdam University Medical Centers, Department of Medical Psychology, University of Amsterdam, Amsterdam, the Netherlands*
3. *Amsterdam University Medical Centers, Medical Library, University of Amsterdam, Amsterdam, the Netherlands.*

Corresponding author: H.W.M. van Laarhoven, h.vanlaarhoven@amsterdamumc.nl
